# Potential Development of Tumor-Targeted Oral Anti-Cancer Prodrugs: Amino Acid and Dipeptide Monoester Prodrugs of Gemcitabine

**DOI:** 10.3390/molecules22081322

**Published:** 2017-08-10

**Authors:** Yasuhiro Tsume, Adam J. Drelich, David E. Smith, Gordon L. Amidon

**Affiliations:** Department of Pharmaceutical Science, College of Pharmacy, University of Michigan, 428 Church Street, Ann Arbor, MI 48109-1065, USA; ytsume@umich.edu (Y.T.); ajdrelic@umich.edu (A.J.D.); smithb@med.umich.edu (D.E.S.)

**Keywords:** gemcitabine prodrugs, stroma cells, pancreatic cancer, metabolism, cytosine deaminase

## Abstract

One of the main obstacles for cancer therapies is to deliver medicines effectively to target sites. Since stroma cells are developed around tumors, chemotherapeutic agents have to go through stroma cells in order to reach tumors. As a method to improve drug delivery to the tumor site, a prodrug approach for gemcitabine was adopted. Amino acid and dipeptide monoester prodrugs of gemcitabine were synthesized and their chemical stability in buffers, resistance to thymidine phosphorylase and cytidine deaminase, antiproliferative activity, and uptake/permeability in HFF cells as a surrogate to stroma cells were determined and compared to their parent drug, gemcitabine. The activation of all gemcitabine prodrugs was faster in pancreatic cell homogenates than their hydrolysis in buffer, suggesting enzymatic action. All prodrugs exhibited great stability in HFF cell homogenate, enhanced resistance to glycosidic bond metabolism by thymidine phosphorylase, and deamination by cytidine deaminase compared to their parent drug. All gemcitabine prodrugs exhibited higher uptake in HFF cells and better permeability across HFF monolayers than gemcitabine, suggesting a better delivery to tumor sites. Cell antiproliferative assays in Panc-1 and Capan-2 pancreatic ductal cell lines indicated that the gemcitabine prodrugs were more potent than their parent drug gemcitabine. The transport and enzymatic profiles of gemcitabine prodrugs suggest their potential for delayed enzymatic bioconversion and enhanced resistance to metabolic enzymes, as well as for enhanced drug delivery to tumor sites, and cytotoxic activity in cancer cells. These attributes would facilitate the prolonged systemic circulation and improved therapeutic efficacy of gemcitabine prodrugs.

## 1. Introduction

Gemcitabine, 2′,2′-difluoro-2′-deoxycytidine, dFdC (Gemzar™) is a cancer drug that is clinically used as a first line treatment for pancreatic cancer and other cancers [1,2]. Gemcitabine is incorporated into DNA synthesis to inhibit cell growth. Like most anticancer drugs, gemcitabine, which is an antimetabolite like floxuridine, is administered intravenously and has a broad spectrum to treat various cancers [3]. However, the adverse effects associated with those chemotherapeutic agents remain severe and many efforts have been made to minimize adverse effects and maximize therapeutic efficacy. Prodrug strategies have been employed to overcome unfavorable physicochemical properties of the drug for the improvement of oral bioavailability and/or the minimization of toxic side effects. Amino acid and dipeptide monoester prodrugs of poorly permeable anticancer and antiviral drugs have been developed and investigated for their improved oral bioavailability and metabolic disposition [4,5,6,7,8,9,10,11,12,13,14,15,16,17,18,19,20,21]. 

Amino acid ester prodrugs and nucleoside analogs have been shown to be substrates for uptake transporters for PEPT1, PEPT2, ATB^0,+^, ENT1, and CNT in the small intestine and other tissues, respectively, and their improved oral bioavailability is attributed by those carrier-mediated mechanisms [22,23,24,25,26,27,28,29,30,31,32,33,34,35,36]. Even though PEPT1 is stereoselective and exhibits greater affinity for l-enantiomers of amino acids than d-enantiomers, PEPT1 still improves the membrane permeability and overall oral absorption of prodrugs with d-amino acids [37,38]. Amino acid monoester prodrugs like valacyclovir significantly enhanced the oral bioavailability of their parent, acyclovir, and several reports suggest that absorption improvement was attributed by oligopeptide transporters [35,36,39,40]. Nucleoside analogs like gemcitabine and floxuridine are mainly transported through nucleoside transporters into the cell, and those transporters are highly expressed in tumor tissues [1,28]. Amino acid ester prodrugs of nucleotide analogs may facilitate enhanced delivery to pancreatic ductal cancer cells such as Panc-1 and Capan-2, since these cells express potential target transporters at high levels [28,41]. 

Gemcitabine is systematically converted to triphosphated gemcitabine by enzymes after being transported into cells and inhibits DNA synthesis [3]. However, gemcitabine is rapidly converted to dFdU in many tissues, including the liver, by the enzyme cytidine deaminase (CDA), while floxuridine, which has similar structure to gemcitabine, is rapidly converted to 5-fluorouracil (5-FU) by the enzyme thymidine phosphorylase (TP) [28,42,43,44]. As a consequence, higher doses of nucleoside analogs are required to display clinical efficacy, which leads to greater toxicity. Therefore, protection of the deamination and glycosidic bond of gemcitabine and floxuridine is required to maintain the high potency of these drugs and to direct the robust inhibition of DNA synthesis. Improving the chemical and enzymatic stability of nucleoside analogs to CDA and TP by prodrug approaches could enhance its therapeutic efficacy and obviate toxicity concerns. 

Although amino acid monoester prodrugs of gemcitabine have exhibited enhanced PEPT1-mediated transport, as well as enzymatic activation in pancreatic cancer surrogate cell systems, gemcitabine prodrugs have to improve their stability because they still need to cross a number of tissue barriers including stromal cells to targeted tumors [16,38,45,46]. d-Amino acid monoester prodrugs of gemcitabine may be delivered to a greater extent at the target site by carrier-mediated transporters and permeated through tumor stroma more than l-amino acid monoester prodrugs due to the elongated systemic circulation time [47,48]. In this report, we describe the stability, membrane permeability, and proliferative activity of gemcitabine prodrugs with stereospecific, l-/d-amino acids, and the dipeptide l-phenylalanyl-l-tyrosine in surrogate cell systems, including the stromal environment. Uptake and permeability of gemcitabine prodrugs were evaluated with HFF cells, a surrogate for stroma cells, to determine the feasibility to deliver drugs to tumors and the advantage/disadvantage of gemcitabine prodrugs over the parent drug gemcitabine. The chemical/enzymatic stability and the enzymatic activation of the prodrugs in Panc-1 and Capan-2 cell homogenates were also evaluated to determine the effects of the amino acid/dipeptide promoiety structure on enzyme-mediated activation. The feasibility of antiproliferative action of selective amino acid/dipeptide gemcitabine prodrugs was also explored using cancer cells that overexpress PEPT1 as well as non-tumoral epithelial cells (MDCK cells) to evaluate the possibility of improved therapeutic efficacy at tumor sites. 

## 2. Results

### 2.1. Chemical and Enzymatic Stability Studies

**Figure 1:**
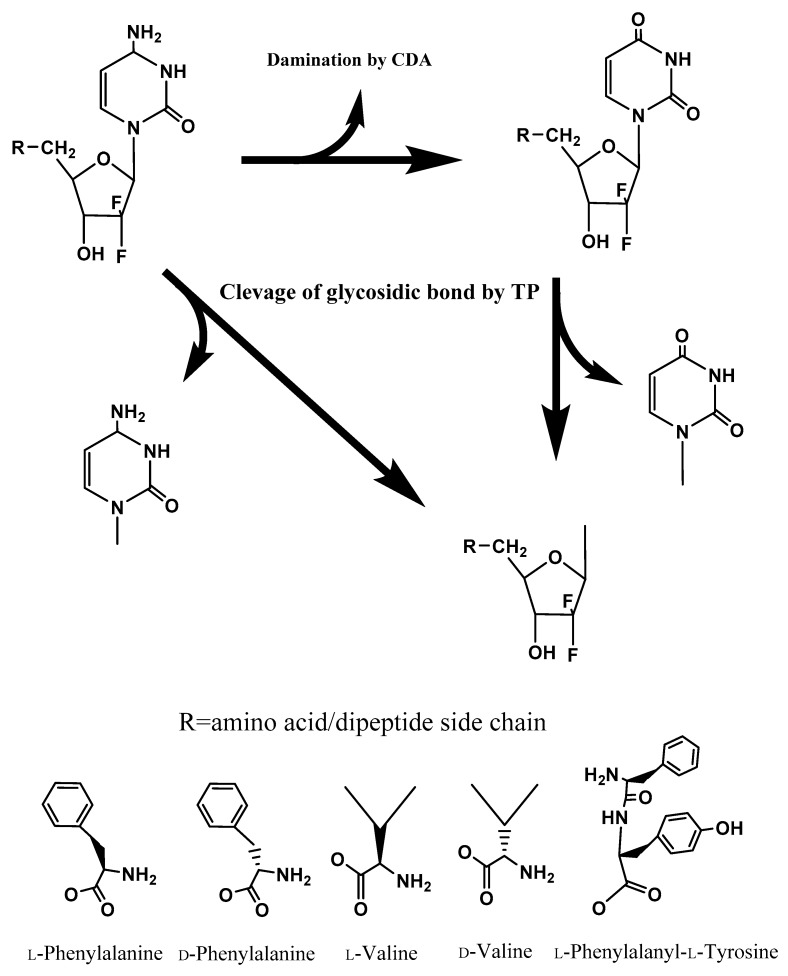
The metabolic pathway of gemcitabine and gemcitabine prodrugs with relevant enzymes.

The experiments concerning prodrug stability were performed at 37 °C in pH 7.4 phosphate buffer, in HFF and MDCK cell homogenates, and in pancreatic cancer cell homogenates, Panc-1 and Capan-2 cells. The estimated half-lives (t_1/2_) obtained from linear regression of pseudo-first-order plots of prodrug concentration vs. time for Gem prodrugs in pH 7.4 phosphate buffers alone, and in MDCK, Panc-1, and Capan-2 cell homogenates, are listed in Table 1 along with previously reported results for the comparison purpose. No significant degradation of Gem and gemcitabine prodrugs was observed in HFF cell homogenate over 120 min (data not shown). Prodrug metabolites such as Gem and cytosine were monitored along with prodrug disappearance in this experiment. However, mass balance was not achieved because cytosine was metabolized even further (Figure 1). 5′-d-valyl-gemcitabine exhibited the highest stability in all media tested and 5′-l/d-valyl-gemcitabine did not metabolize in MDCK cells. The dipeptide prodrug, 5′-l-phenylalanyl-tyrosyl-gemcitabine, was chemically less stable compared to amino acid prodrugs, due to the possible formation of diketopiperazine by a dipeptide promoiety, and exhibited 4-fold faster metabolism/activation in cell homogenates compared to one in buffer (pH 7.4). All prodrugs exhibited 4- to 87-fold shorter half-lives in cell homogenates than in pH 7.4 phosphate buffer, suggesting enzyme-catalyzed hydrolysis. The composition of the amino acids in 5′ position exerted a profound effect on the stability of the ester bond, especially gemcitabine prodrugs, with an unnatural form (d-) of amino acid enzymatically more stable than one with natural form (l-) of amino acid (Table 1).

### 2.2. Thymidine Phosphorylase Activity against Gemcitabine and Gemcitabine Prodrugs

The metabolic stability of Gem and Gem prodrugs was assessed using the pure enzyme, thymidine phosphorylase. The results shown in Table 2 indicate that Gem was rapidly degraded to the less active metabolite, cytosine, by thymidine phosphorylase. The amino acid and dipeptide monoester prodrugs of Gem were found to be quite resistant to degradation by thymidine phosphorylase. Gem prodrugs were, at least, 5- to 10-fold more stable than Gem to degradation by thymidine phosphorylase. The half-lives of 5′-d-phenylalanyl-gemcitabine, 5′-l-valyl-gemcitabine, and 5′-d-valyl-gemcitabine were in excess of 120 min, reflecting their superior resistance to metabolic degradation by thymidine phosphorylase.

### 2.3. Cytidine Deaminase Activity against Gemcitabine and Gemcitabine Prodrugs

The metabolic stability of Gem and Gem prodrugs was assessed using the pure enzyme, CDA. The results shown in Table 3 indicate that Gem was rapidly deaminated to the less active metabolite, dFdU, by CDA. The amino acid and dipeptide monoester prodrugs of Gem were found to be quite resistant to deamination by CDA. Gem prodrugs were, at least, 15-fold more stable than Gem to degradation by CDA. The half-lives of 5′-d-phenylalanyl-gemcitabine, 5′-l-valyl-gemcitabine, 5′-d-valyl-gemcitabine, and 5′-l-phenylalanyl-l-tyrosyl-gemcitabine were in excess of 120 min, reflecting their superior resistance to metabolic degradation by CDA.

### 2.4. [^3^H]Gly-Sar Uptake Inhibition

The IC_50_ values of Gem and Gem Prodrugs for the intake transporters, PEPT1, determined using inhibition of Gly-Sar uptake in Caco-2 cells, are summarized in Table 4. All Gem prodrugs exhibited greater affinity for the intake transporter than their parent, Gem. Gem exhibited minimal inhibitory activity of Gly-Sar uptake into Caco-2 cells and the IC_50_ value was 26.8 ± 9.3 mM. All gem prodrugs exhibited an 8- to 38-fold better IC_50_ value than Gem. Among them, 5′-l-phenylalanyl-gemcitabine exhibited the highest affinity (IC_50_ value 0.7 ± 0.3 mM). The promoieties (phenylalanyl- and valyl-) with an unnatural amino acid (d-) exhibited slightly lower affinity to transporters than the corresponding promoieties with a natural amino acid (l-).

### 2.5. Uptake Study of Gem and Gem Prodrugs in HFF Cells

**Figure 2:**
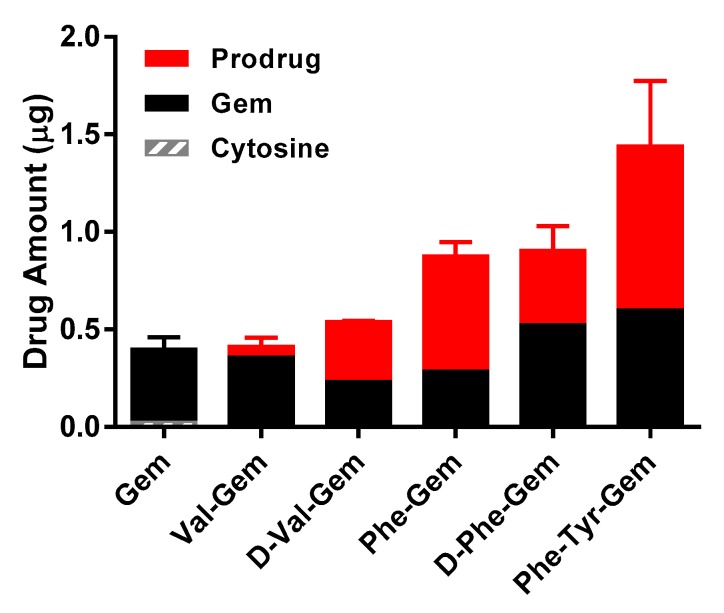
Uptake study of Gem and Gem prodrugs in HFF cells (Mean ± S.D., *n* = 4).

The uptake of mono amino acid/dipeptide monoester prodrugs of Gem and parent, Gem, was determined at 37 °C in HFF cell. The uptake amount of all Gem prodrugs in HFF cells was 1.1- to 3.8-fold higher than their parent, Gem (Figure 2). The amounts of Gem prodrugs in HFF cells correlated with their logP values (Table 5).

### 2.6. HFF Cell Permeability of Gem and Gem Prodrugs

The apical-to-basolateral permeability of mono amino acid/dipeptide monoester prodrugs of Gem and parent, Gem, was determined at 37 °C in HFF cell monolayers. Table 6 shows the permeability values. With the exception of 5′-l-valyl-gemcitabine, the permeability of all Gem prodrugs across HFF monolayers was 2- to 4-fold higher than their parent, Gem. All prodrugs displayed better membrane permeability in the HFF cell monolayer.

### 2.7. Cell Proliferation Assays

GI_50_ values for Gem and 5′-mono amino acid/dipeptide monoester prodrugs of Gem were determined in cell proliferation studies with the pancreatic cancer cell lines, Panc-1 and Capan-2, as shown in Table 7. All prodrugs exhibit enhanced antiproliferative activity in the three cell lines compared to parent Gem. Thus, the GI_50_ values of all Gem prodrugs were in the range of 2.8–7.6 mM in Panc-1 and Capan-2 cells, as opposed to the GI_50_ value of 8.5 mM for Gem in Capan-2 cells. The antiproliferative activity of Gem was not observed in Panc-1 cells. These results are consistent with trends observed in antiproliferative activity in AsPC-1 cells [38]. Interestingly, 5′-l-valyl-gemcitabine, 5′-d-valyl-gemcitabine, and 5′-l-phenylalanyl-l-tyrosyl-gemcitabine did not exhibit any inhibitory effect while 5′-l-phenylalanyl-gemcitabine, 5′-d-phenylalanyl-gemcitabine, and gemcitabine did display antiproliferative activity in MDCK cells.

## 3. Discussion

Prodrug approaches, such as a parent drug coupled with an amino acid, have been widely employed to improve the intestinal absorption and oral bioavailability of poorly permeable drugs. The antivirals valacyclovir and valganciclovir [27,49] are the commercially and clinically successful examples of prodrug strategies with amino acids. The improved oral bioavailability of these prodrugs [40,50] has been attributed to their enhanced transport by oligopeptide transporters in the small intestine like Pept1 [5,35,36,39,50,51]. Following intestinal absorption, those prodrugs would then be efficiently converted to the parent drug by valacyclovirase [52,53]. A variety of substrates for the Pept1 transporter have been investigated to understand substrate suitability and design for further approaches [22,23,46,54,55,56,57,58,59]. We have reported the prodrug synthesis and its evaluation of mono amino acid and the dipeptide monoester prodrugs of antiviral and anticancer drugs such as floxuridine, gemcitabine, melphalan, acyclovir, oseltamivir, and 2-bromo-5,6-dichloro-1-(β-d-ribofuranosyl)benzimidazole (BDCRB) [5,6,9,10,11,14,15,16,17,18,19,20,38,39,60,61,62]. In this report, we describe the chemical and enzymatic stability of gemcitabine prodrugs, their affinity to transporters in Caco-2 cells, the cellular uptake and transport in HFF cells (a surrogate for stroma cell transport), and antiproliferative activity in two pancreatic duct cell lines, Panc-1 and Capan-2, along with MDCK cells. The studies demonstrated that those mono amino/dipeptide monoester prodrugs generally displayed an enhanced affinity to the PEPT1 transporter, a range of bioactivation rates, and protection of the glycosidic bond to metabolic enzymes such as thymidine phosphorylase and cytidine deaminase. Major findings of this study are: (1) enzymes had more specific stereospecificity for amino acid prodrug esters than transporters, and the promoiety with unnatural form (d-) amino acids exhibited enhanced enzymatic stability. Gemcitabine prodrugs showed resistance against the enzymes TP and CDA to maintain their prodrug structures, and the 5′- position of promoieties was more effective than the 3′- position; (2) amino acid/dipeptide monoester prodrugs of gemcitabine had better affinity to uptake transporters than their parent Gem and had better membrane permeability in HFF cells, indicating that gemcitabine prodrugs had the potential to reach tumor sites through stroma cells more than their parent; and (3) gemcitabine prodrugs displayed better antiproliferative activity than their parent in pancreatic cancer cells but did not exhibit cytotoxicity growth in the non-tumor cell line, MDCK cells, suggesting that the toxicity of gemcitabine prodrugs was absent or minimal, and gemcitabine prodrugs had the potential to improve the therapeutic efficacy, suggesting the necessity of prodrug activation. 

The dipeptide prodrug was chemically less stable in pH 7.4 buffer than the mono amino acid monoester prodrugs, regardless of the stereochemistry of the amino acids. Since no degradation of mono amino monoester prodrugs was observed, the dipeptide monoester prodrug likely degraded *via* parallel pathways similar to those suggested for Gly-Phe dipeptide alkyl ester prodrugs by Larsen and colleagues [63]. Additionally, a diketopiperazine cyclization product is also possible due to intramolecular condensation of the ester group with the free amino group of the dipeptide monoester prodrug. This intramolecular aminolysis has been reported, and this reaction would be negligible at pH values below 6 [63,64,65]. Indeed, the dipeptide monoester prodrugs were stable, and the formation of diketopiperizine was not observed at pH values below 6 [16,18]. 

The enzymatic stability of 5′-d-phenylalanyl-gemcitabine and 5′-d-valyl-gemcitabine was significantly enhanced compared to prodrugs with the same amino acid (l-) promoiety, suggesting that the enzymes have high affinity in their substrate specificity and that unnatural form (d-) amino acids protect the enzyme-catalyzed hydrolysis of the ester linkage. Gemcitabine was quickly metabolized in the pancreatic cancer cell homogenate of Capan-2 cells, but substantial metabolism of gemcitabine was not observed in the pancreatic cancer cell homogenate of Panc-1 cells, suggesting different enzyme expression profiles. All prodrugs displayed resistance to TP and CDA but their parent, gemcitabine, was quickly metabolized. 3′-l-Valyl-gemcitabine also exhibited resistance to TP but the resistance was not as effective as 5′-l-valyl-gemcitabine, suggesting that the 5′-site promoiety would have structural hindrance to protect the glycosidic bond, but the 3′-site promoiety would not have structural hindrance as strong as the 5′-site (Table 2 and Table 3).

Results of affinity studies of Gem and Gem prodrugs in Caco-2 cells were consistent with the previous report of floxuridine, which has a similar chemical structure to Gem and floxuridine prodrugs [9,17,19]. The transporter affinities of prodrugs with an unnatural amino acid were lower than ones with a natural amino acid (Table 4). Since, unlike Caco-2 cells, the protein expression of transporters in foreskin fibroblasts (HFF) cells is low, the prodrugs would permeate HFF cell monolayers by simple diffusion [66,67,68]. The results of uptake studies with gemcitabine prodrugs correlate with logP values of test drugs (Table 5 and Figure 2). The uptake amounts and permeabilities of the gemcitabine prodrugs with the natural form (l-) of amino acid were consistently lower in HFF cells compared to the prodrugs with their corresponding unnatural form (d-) of amino acid (Figure 2 and Table 6). Since 5′-valyl-gemcitabine and 5′-phenylalanyl-gemcitabine should have the same LogP values regardless of the stereochemistry of amino acid (l-/d-), and those prodrugs exhibited different uptake amounts and different prodrug/parent drug ratios, it is suggested that the cellular accumulation of prodrugs with the natural form of amino acids are metabolized to a greater extent than prodrugs with the unnatural form of amino acids. Indeed, the prodrugs with the unnatural form of amino acids maintained more prodrug, relative to hydrolyzed drug, than ones with the natural form amino acids (Figure 2 and Table 1). Overall, these findings indicate an improvement of those prodrugs over parent drug in both stability and permeability characteristics. 

Cell proliferation studies in pancreatic duct cancer cell lines confirmed the enhanced potency of the amino acid/dipeptide monoester prodrugs compared to the parent gemcitabine. The activation/metabolism of 5′-d-valyl-gemcitabine in MDCK, Panc-1, and Capan-2 cells was not observed over 2 h in stability studies, but the prodrug was activated and exhibited antiproliferative activity in only Panc-1 and Capan-2 cells during 24 h. Especially in Panc-1 cells, all prodrugs exhibited tumor growth inhibition, while their parent drug, gemcitabine, did not exhibit any inhibitory activities (Table 7). Those pancreatic cancer cells that highly expressed the Pept1 transporter and amino acid/dipeptide monoester prodrugs of chemotherapeutic agents exhibited affinity for this uptake transporter [16,17,18,19,38,62]. Therefore, prodrugs are apparently delivered to a greater degree into those cancer cells, while gemcitabine is delivered to a lesser degree into the cells. Even though gemcitabine is delivered into the cancer cells, gemcitabine is quickly metabolized to non-toxic metabolites like cytosine by thymidine phosphorylase and other enzymes, which would reduce the antiproliferative activity in pancreatic cancer cells [69,70,71]. l-Amino acid monoester prodrugs exhibited slightly better GI_50_ values than d-amino acid monoester prodrugs in Panc-1 cells, while d-amino acid monoester prodrugs exhibited slightly better GI_50_ values than l-amino acid monoester prodrugs in Capan-2 cells, thereby suggesting that varying enzyme expression levels and species differences exist between those two pancreatic cancer cells. The GI_50_ values of gemcitabine prodrugs did not exhibit any discernible correlations with their permeability and/or bioactivation profiles in these cells. The different amino acid and stereoisomer promoieties of prodrugs would contribute to the different rates of prodrug activation inside cancer cells by particular activation enzymes. Therefore, it would be difficult to discern a meaningful correlation between GI_50_ values and prodrug uptake/permeabilities with limited experimental data. 

The intracellular anabolism of gemcitabine prodrugs may illustrate that transported drugs and prodrugs are converted to gemcitabine and cytosine, or dFdU and further metabolites, via a sequential enzymatic pathway with higher concentrations of TP in tumor tissue and the ubiquitous presence of CDA (Figure 1). Taken together, our results indicate that the unnatural form amino acid monoester prodrugs of gemcitabine exhibit the potential for improved oral absorption, improved delivery into tumor sites, and enhanced antiproliferative activity. Therefore, gemcitabine prodrugs with unnatural forms of amino acid might possess advantages over ones with the natural form of amino acid for cancer target delivery, and potentially oral target delivery. Delayed enzymatic activation and enhanced metabolic resistance, along with oligopeptide transporter affinity, may facilitate the prolonged systemic circulation and enhanced therapeutic action of gemcitabine prodrugs. By demonstrating enhanced stability in biological surrogate media and cell homogenates, gemcitabine prodrugs with unnatural amino acids have potential to be developed as oral drug products with targeting to cancer cells. 

## 4. Materials and Methods

### 4.1. Materials

Gemcitabine (Gem) was extracted from the lyophilized powder (Gemzar™) supplied by Eli Lilly Pharmaceuticals (Indianapolis, IN, USA). The tert-butyloxycarbonyl (Boc) protected amino acids Boc-l-valine, Boc-l-phenylalanine, Boc-d-valine, Boc-d-phenylalanine, and Boc-l-phenylalanyl-l-tyrosine were obtained from Chem-Impex (Wood Dale, IL, USA). High-performance liquid chromatography (HPLC) grade acetonitrile was obtained from Fisher Scientific (St. Louis, MO, USA). *N,N*-dicyclohexylcarbodiimide (DCC), *N,N*-dimethylaminopyridine (DMAP), trifluoroacetic acid (TFA), and all other reagents and solvents were purchased from Aldrich Chemical Co. (Milwaukee, WI, USA). Cell culture reagents were obtained from Invitrogen (Carlsbad, CA, USA) and cell culture supplies were obtained from Corning (Corning, NY, USA) and Falcon (Lincoln Park, NJ, USA). All chemicals were either analytical or HPLC grade. 

### 4.2. Gemcitabine Prodrug Synthesis

The synthesis and characterization of 5′-mono amino acid and dipeptide monoester prodrugs of Gem have been reported previously and synthesis was used similar to synthase Gem prodrugs [9,10,17,19,20,29]. Briefly, Boc-protected amino acid or dipeptide, (1.1 mmol), DCC (1.1 mmol), and DMAP (0.1 mmol) were allowed to react with Gem (1 mmol) in 7 mL of dry DMF for 24 h. The reaction progress was monitored by TLC (ethyl acetate). The reaction mixture was filtered and DMF was removed under vacuum at 40 °C. The residue was extracted with ethyl acetate (30 mL), and washed with water (2 × 20 mL) and saturated NaCl (20 mL). The organic layer was dried over MgSO_4_ and concentrated under vacuum. The reaction yielded a mixture of 3′-monoester, 5′-monoester, and 3′,5′-diester Gem prodrugs. The three spots observed on TLC were separated and purified using column chromatography (dichloromethane (DCM)/methanol, 20:1). Fractions from each spot were concentrated under vacuum separately. The Boc group was cleaved by treating the residues with 5 mL TFA:DCM (1:1). After 4 h, the solvent was removed and the residues were reconstituted with water and lyophilized. The TFA salts of amino acid prodrugs of Gem were obtained as white fluffy solids. The combined yield of Gem prodrugs was ~60%. HPLC was used to evaluate the prodrug purity. Prodrugs were between 90–99% pure. These prodrugs were easily separated from parent drug by HPLC. Electrospray ionization mass spectra (ESI-MS) were obtained on a Micromass LCT ESI-MS. The observed molecular weights of all prodrugs were found to be consistent with that required by their structure. The structural identity of the prodrugs was then confirmed using proton nuclear magnetic resonance spectra (^1^H-NMR). ^1^H-NMR spectra were obtained on a 300 MHz Bruker DPX-300 NMR spectrometer (Billerica, MA, USA). 

### 4.3. Cell Culture

Panc-1 cells (passages 22–30) and Capan-2 cells (passages 31–36) from American Type Culture Collection (Rockville, MD, USA) were routinely maintained in RPMI-1640 containing 10% fetal bovine serum. HFF cells (passages 15–26) and MDCK cells (passages 25–32) from American Type Culture Collection (Rockville, MD, USA) were routinely maintained in DMEM containing 10% fetal bovine serum, 1% nonessential amino acids, 1 mmol/L sodium pyruvate, and 1% l-glutamine at 5% CO_2_ and 90% relative humidity at 37 °C. Cells were grown in antibiotic-free media to avoid possible transport interference by antibiotics.

### 4.4. Hydrolysis Studies

*Enzymatic Stability*. Confluent HFF, MDCK, Panc-1, and Capan-2 cells were rinsed with saline twice. The cells were washed with 5 mL of pH 7.4 phosphate buffer (10 mmol/L), lysed by ultrasonication (Micro ultrasonic cell disrupter Model KT40, Kontes, Vineland, NJ, USA), and pelleted by centrifugation for 5 min at 1000× *g*. The protein amount was quantified with Bio-Rad (Hercules, CA, USA) DC Protein Assay using bovine serum albumin as a standard. The protein amount was adjusted to 500 μg/mL and the hydrolysis reactions were carried out in 96-well plates (Corning). HFF, MDCK, Panc-1, and Capan-2 cell suspensions (250 µL) were placed in triplicate wells, the reactions started with the addition of substrate, and cells were incubated at 37 °C for 120 min. At the desired time point, sample aliquots (35 µL) were removed and added to 150 µL of acetonitrile (ACN) containing 0.1% TFA. The mixtures were filtered with a 0.45 µm filter at 1000× *g* for 10 min at 4 °C. The filtrate was then analyzed via reverse-phase HPLC. 

*Chemical stability.* The nonenzymatic hydrolysis of the prodrugs was determined as described above, except that each well contained pH 7.4 phosphate buffer (10 mmol/L) instead of cell homogenate or human plasma. 

*Resistance to metabolism of gemcitabine and its prodrugs by thymidine phosphorylase.* The stability of Gem and Gem prodrugs in the presence of thymidine phosphorylase (TP) was assessed by incubating the desired substrates (200 µM) with TP (2.0 ng/µL) in phosphate buffer (pH 7.0) at 37 °C. Aliquots of the incubation mixture were sampled at 0, 1, 3, 5, 10, 30, 60, and 120 min, and quenched with cold acetonitrile (ACN) with 0.1% TFA, filtered through 0.45 µm membrane, and analyzed for the concentrations of Gem prodrugs and Gem by HPLC.

*Resistance to metabolism of gemcitabine and its prodrugs by cytidine deaminase.* The stability of Gem and Gem prodrugs in the presence of cytidine deaminase (CDA) was assessed by incubating the desired substrates (200 µM) with CDA (5.0 ng/µL) in phosphate buffer (pH 6.5) at 37 °C. Aliquots of the incubation mixture were sampled at 0, 3, 5, 10, 30, 60, and 120 min, and quenched with cold acetonitrile (ACN) with 0.1% TFA, filtered through 0.45 µm membrane, and analyzed for the concentrations of Gem prodrugs and Gem by HPLC.

### 4.5. [^3^H]Gly-Sar Uptake Inhibition

Caco-2 cells at 10 days postseeding were incubated with 10 µmol/L Gly-Sar (9.98 µmol/L Gly-Sar and 0.02 µmol/L [^3^H]Gly-Sar (Moravek Biochemicals, Brea, CA, USA)), along with various concentrations (5–0.05 mmol/L) of Gem and Gem prodrugs for 30 min. After the incubation, the drug solution was removed. The cells were gently washed three times with ice-cold PBS and solubilized with 2 mL of scintillation cocktail (ScintiVerse, Fisher Scientific, St. Louis, MO, USA), and the radioactivity was determined by scintillation counting (Beckman LS-9000, Beckman Instruments, Fullerton, CA, USA). Inhibitory concentration 50 (IC_50_) values were determined using nonlinear data fitting (GraphPad Prism version 6, GraphPad Software, Inc., La Jolla, CA, USA).

### 4.6. Uptake Study of Gemcitabine Prodrugs and Gemcitabine in HFF Cells

HFF cells were grown on a 12-well plate for 24 days. Wells were rinsed with MES (pH 6.0) buffer twice. Fresh MES buffer was reapplied to each well and incubated at 37 °C for 15 min. Each drug was individually tested from freshly prepared solutions in MES buffer (0.1 mM, total 0.3 mL). The solution was placed in each well and incubated at 37 °C for 30 min. Drug solution was removed and 3 mL of ice-cold PBS was immediately placed in each well. Each well was rinsed with 3 mL of cold-PBS twice and 0.5 mL of methanol:H_2_O (1:1) containing 0.1% TFA was placed in each well. The cell suspension was collected and transferred to a new tube. Those tubes were spun at 1000× *g* at 4 °C for 5 min. The supernatant was mixed with an equal amount of water with either 0.1% formic acid or 0.1% ammonium hydroxide for HPLC analysis. The cell pellets were used to determine the protein amount with the Bio-Rad (Hercules, CA, USA) DC Protein Assay using bovine serum albumin as a standard. 

### 4.7. Transepithelial Transport Studies in HFF Cells

HFF cell monolayers were grown on collagen-coated polytetrafluoroethylene membranes for 28 days. Transepithelial electrical resistance (TEER) was monitored and values above 180 Ω/cm^2^ in HFF cells were used in the study. Apical and basolateral sides of the transwell inserts were washed with MES (pH 6.0) and HEPES (pH 7.4), respectively. Fresh MES and HEPES buffers were reapplied to transwell inserts and incubated at 37 °C for 15 min. A freshly prepared 0.1 mM drug solution in MES buffer was placed in the donor chamber and the receiver chamber was filled with HEPES buffer. The volumes of donor and receiver chambers were 0.5 mL and 1.5 mL, respectively. The area of the exposed monolayer was 1.12 cm^2^. Sampling from the receiver chamber (100 µL) was conducted up to a period of 2 h at 15, 30, 45, 60, 75, 90, 105, and 120 min, at 37 °C, and replaced with an equal volume of fresh HEPES buffer to maintain sink conditions in the receiver chamber. All samples were immediately acidified with 0.1% TFA and analyzed by HPLC. 

### 4.8. Data Analysis

The initial rates of hydrolysis were used to obtain the apparent first-order rate constants and subsequent half-lives. The apparent first-order degradation rate constants of various Gem prodrugs at 37 °C were determined by plotting the logarithm of the prodrug remaining as a function of time. The slopes of these plots are related to the rate constant, k, and given by

k = 2.303 × slope (log C vs. time)(1)

The degradation half-lives were then calculated by the equation,

t_1/2_ = 0.693/k (2)

Statistical significance was evaluated with GraphPad Prism version 6.0 (La Jolla, CA, USA) by performing a one-way analysis of variance with post-hoc Tukey’s test to compare means.

The apparent permeability (*P_app_*) for the prodrugs was calculated using the following equations:

*Flux* = *J_ss_* = *dM/dt* (3)

where *J_ss_* is the steady state flux, *M* is the cumulative amount of prodrug, and regenerated mono amino acid prodrug, drug, and cytosine is in the receiver compartment. The concentrations of Gem and Gem prodrugs in the receiver and donor compartments were analyzed using HPLC. 

### 4.9. HPLC Analysis

The concentrations of prodrugs and their metabolites were determined with an Agilent HPLC system (Agilent Technologies, Santa Clara, CA, USA). The HPLC system consisted of Agilent pumps (1100 series), an Agilent autosampler (1200 series), and an Agilent UV-Vis detector (1100 series) controlled by Chemstation^®^ 32 software. Samples were resolved in the Agilent Eclipse XDB-C_18_ reverse-phase column (3.5 µm, 4.6 × 150 mm), equipped with a guard column for gemcitabine and gemcitabine prodrugs. The mobile phase consisted of 0.1% TFA/water (Solvent A) and 0.1% TFA/acetonitrile (Solvent B) with the solvent B gradient changing from 0–56% at a rate of 2%/min during a 14 min run. Standard curves generated for cytosine, Gem, and Gem prodrugs were utilized for the quantitation of the integrated area under peaks. The detection wavelength was 254 nm and 280 nm for drug compounds.

### 4.10. Cell Proliferation Assays

Cell proliferation studies were conducted with MDCK, Panc-1, and Capan-2 cell lines. The cells were seeded onto 96-well plates at 125,000 cells per well and allowed to attach/grow for 24 h before drug solutions were added. The culture medium (RPMI-1640 + 10% fetal bovine serum) was removed and the cells were gently washed once with sterile pH 6.0 uptake buffer. Gem and Gem prodrugs were serially diluted in pH 6.0 uptake buffer from 5 to 0.25 mmol/L. Buffer alone was used as 100% viability control. The wash buffer was removed and 25 µL drug solution per well was added and incubated at 37 °C for 4 h with MDCK, Panc-1, and Capan-2 cells in the cell incubator. After this time period, the drug solutions were removed and the cells were gently washed twice with sterile uptake buffer. The culture medium was then added to each well after washing. The cells were allowed to recover for 24 h before evaluating cell viability via 2,3-bis[2-methoxy-4-nitro-5-sulfophenyl]-2*H*-tetrazolium-5-carboxanilide inner salt (XTT) assays. A mixture (30 µL) containing XTT in sterile RPMI-1640 without phenol red (1 mg/mL) and phenazine methosulfate (*N*-methyl dibenzopyrazine methyl sulfate in sterile PBS, 0.383 mg/mL) reagents were added to the cells and incubated at 37 °C for 1 h for the color to develop. Absorbance readings at 450 nm were recorded. The data were plotted and the GI_50_ values, with a concentration showing 50% of the cell growth inhibitory effect, were calculated using GraphPad Prism version 6.0 (La Jolla, CA, USA) by nonlinear data fitting.

## Figures and Tables

**Table 1:** Stability of gemcitabine prodrugs in pH 7.4 Buffer and pancreatic cancer cell homogenates. (Mean ± S.D., *n* = 3).

Prodrug	Buffer pH 7.4 t_1/2_ (min)	Panc-1 Cell Homogenates t_1/2_ (min)	Capan-2 Cell Homogenates t_1/2_ (min)	MDCK Cell Homogenates t_1/2_ (min)
Gemcitabine	>120 *	>120 #	5.2 ± 2.4	72.9 ± 29.1
*Mono amino acid prodrugs*				
5′-l-Phenylalanyl-gemcitabine	>120 *	9.8 ± 1.2	1.4 ± 0.1	4.8 ± 1.2
5′-d-Phenylalanyl-gemcitabine	>120 *	29.2 ± 5.7	14.2 ± 0.4	94.9 ± 15.7
5′-l-Valyl-gemcitabine	>120 *	23.9 ± 0.7	4.8 ± 1.3	>120
5′-d-Valyl-gemcitabine	>120 *	>120	>120	>120
*Dipeptide prodrugs*				
5′-l-Phenylalanyl-l-tyrosyl-gemcitabine	33.6 ± 1.4	30.2 ± 1.1 #	8.0 ± 2.3	60.2 ± 2.3

* Previously reported in [38]; # Previously reported in [16].

**Table 2:** Stability of gemcitabine and gemcitabine prodrugs in the presence of thymidine phosphorylase (mean ± S.D., *n* = 3).

Prodrug/Drug	t_1/2_ (min)
Gemcitabine	6.0 ± 1.8 *
*Mono amino acid prodrugs*	
5′-l-Phenylalanyl-gemcitabine	31.9 ± 5.0 *
5′-d-Phenylalanyl-gemcitabine	>120 *
5′-l-Valyl-gemcitabine	>120 *
5′-d-Valyl-gemcitabine	>120 *
*Dipeptide prodrugs*	
5′-l-Phenylalanyl-l-tyrosyl-gemcitabine	63.0 ± 0.3
3′-l-Valyl-gemcitabine	56.0 ± 6.9

* Previously reported [38].

**Table 3:** Stability of gemcitabine and gemcitabine prodrugs in the presence of cytidine deaminase (mean ± S.D., *n* = 3).

Prodrug/Drug	t_1/2_ (min)
Gemcitabine	<3
*Mono amino acid prodrugs*	
5′-l-Phenylalanyl-gemcitabine	44.6 ± 18.8
5′-d-Phenylalanyl-gemcitabine	>120
5′-l-Valyl-gemcitabine	>120
5′-d-Valyl-gemcitabine	>120
*Dipeptide prodrugs*	
5′-l-Phenylalanyl-l-tyrosyl-gemcitabine	>120

**Table 4:** [^3^H] Gly-Sar uptake inhibition of gemcitabine and gemcitabine prodrugs in Caco-2 cells (mean ± S.D., *n* = 3).

Prodrug/Drug	IC_50_ Caco-2 (mM)
Gemcitabine	26.8 ± 9.3
*Mono amino acid prodrugs*	
5′-l-Phenylalanyl-gemcitabine	0.7 ± 0.3
5′-d-Phenylalanyl-gemcitabine	3.4 ± 0.7
5′-l-Valyl- gemcitabine	1.6 ± 0.6
5′-d-Valyl- gemcitabine	2.8 ± 1.1
*Dipeptide prodrugs*	
5′-l-Phenylalanyl-l-tyrosyl-gemcitabine	1.6 ± 0.3

**Table 5:** Amino acid ester prodrugs of gemcitabine.

Prodrug	LogP ^a^
5′-l/d-Valyl-gemcitabine	−0.37
5′-l/d-Phenylalanyl-gemcitabine	0.42
5′-l-Phenylalanyl-l-tyrosyl-gemcitabine	1.04

^a^ Calculated using ChemDraw.

**Table 6:** Apparent permeability coefficients (*P_app_*) of gemcitabine and monoester prodrugs of gemcitabine in the apical-to-basolateral direction across HFF monolayers (mean ± S.D., *n* = 3).

Prodrug/Drug	*P_app_*, HFF (×10^−6^ cm/s)
Gemcitabine	1.6 ± 0.1
*Mono amino acid prodrugs*	
5′-l-Phenylalanyl-gemcitabine	2.7 ± 0.3
5′-d-Phenylalanyl-gemcitabine	3.9 ± 0.4
5′-l-Valyl-gemcitabine	1.7 ± 0.3
5′-d-Valyl-gemcitabine	5.1 ± 1.0
*Dipeptide prodrugs*	
5′-l-Phenylalanyl-l-tyrosyl-gemcitabine	5.1 ± 0.3

**Table 7:** Cell growth inhibition in Panc-1, Capan-2, and MDCK cells (mean ± S.D., *n* = 3–6).

Prodrug/Drug	GI_50_ Panc-1 (mM)	GI_50_ Capan-2 (mM)	GI_50_ MDCK (mM)
Gemcitabine	None *	8.5 ± 0.2	58.9 ± 3.8
*Mono amino acid prodrugs*			
5′-l-Phenylalanyl-gemcitabine	2.8 ± 0.1	7.6 ± 2.3	7.1 ± 2.9
5′-d-Phenylalanyl-gemcitabine	3.0 ± 0.1	6.3 ± 2.0	79.3 ± 3.4
5′-l-Valyl- gemcitabine	3.0 ± 0.3	5.8 ± 0.6	None
5′-d-Valyl- gemcitabine	3.6 ± 0.2	5.5 ± 0.8	None
*Dipeptide prodrugs*			
5′-l-Phenylalanyl-l-tyrosyl-gemcitabine	3.2 ± 0.7 *	3.6 ± 1.3	None

None—No inhibitory activity detected. * Previously reported in [16].
